# Uncovering Care Challenges for Head and Neck Cancer Patients and Caregivers in the United States: A Scoping Review

**DOI:** 10.7759/cureus.80774

**Published:** 2025-03-18

**Authors:** Sharon Amole, Roger M Roe, Soroush Farsi, Dang-Khoa Nguyen, Emily Hallgren, Lauren Tong, Deanne King

**Affiliations:** 1 Department of Otolaryngology – Head and Neck Surgery, University of Arkansas for Medical Sciences, Little Rock, USA; 2 Department of Internal Medicine, University of Arkansas for Medical Sciences, Fayetteville, USA

**Keywords:** cancer patients, caregivers mental health, caregiver strain, head and neck cancer surgery, head and neck neoplasms

## Abstract

Individuals undergoing treatment for head and neck cancer (HNC) face numerous challenges, including financial and food insecurity, which affect both the patients and their caregivers. This scoping review explores and consolidates the current literature addressing the care challenges experienced by HNC patients and their caregivers. Eligible studies were identified through a search of MEDLINE and Embase databases. The scoping review was conducted in accordance with Arksey and O'Malley's five-stage methodology and followed the Preferred Reporting Items for Systematic Reviews and Meta-Analyses Extension for Scoping Reviews (PRISMA-ScR) guidelines. Data extraction and analysis were performed using a thematic approach.

The inclusion criteria encompassed full-text articles published in English that examined challenges faced by both adult HNC patients and caregivers as primary outcomes in the United States. Eligible studies underwent assessment using the Covidence data screening and extraction tool. Information encompassing general study details, article characteristics, and scoping review-relevant details were extracted through Research Electronic Data Capture (REDCap).

Out of the 822 articles initially identified, 40 met the inclusion criteria. The majority of these comprised descriptive surveys (n = 23; 54%), retrospective chart reviews (n = 7; 18%), cross-sectional studies (n = 6; 15%), and prospective cohort studies (n = 13). Within the included studies, the majority of the studies (n = 36) concentrated on quantifying and describing the financial toxicity and out-of-pocket costs experienced by patients with HNC. Two articles concentrated on challenges associated with providing nutritional care to patients with HNC. Additionally, four studies explored the quality of life and the burden experienced by caregivers of patients undergoing treatment for HNC.

Despite the existing body of literature addressing the financial challenges faced by patients with HNC, a noticeable gap exists in the literature concerning challenges related to providing nutritional care and the obstacles faced by caregivers in delivering care to this population. This scoping review offers a comprehensive overview of these challenges and the literature's current focus on addressing them. The findings emphasize the necessity for further research to gain a deeper understanding of the experiences of both patients and caregivers, evaluate the impact of interventions, and bridge knowledge gaps. Ultimately, such endeavors will contribute to enhancing the quality of care and support for individuals affected by HNC.

## Introduction and background

Head and neck cancer (HNC) presents unique and significant challenges for both patients and caregivers [1,2]. HNC ranks as the seventh most prevalent type of cancer worldwide and encompasses a heterogeneous spectrum of malignancies originating from the anatomic regions comprising the upper aerodigestive tract [3,4]. HNC accounts for around 4% of all cancers in the United States. In 2023, an estimated 66,920 people (49,190 men and 17,730 women) were diagnosed with HNC [5]. The diagnosis, treatment, and post-treatment phases of HNC can impose significant physical, emotional, and financial burdens on patients and caregivers [6]. These challenges often necessitate caregivers to master complex technical skills, such as tracheostomy and percutaneous endoscopic gastrostomy tube care [7].

Certain populations are particularly susceptible to HNC, with higher rates observed among men, older adults, and individuals of lower socioeconomic status [8]. HNC puts these populations at a greater risk for financial insecurity as a significant portion of the patients exit the workforce. This insecurity can further exacerbate negative psychological responses, leading to a loop of insults [9]. Additionally, these financial hardships can include external expenses alongside out-of-pocket medical healthcare bills [10]. Among the other issues faced by HNC patients and their respective caregivers is psychological burden of receiving and providing care. Many HNC patients suffer from negative psychological and self-esteem changes as a result of unmet needs, lack of community support, and diminished quality of life. These negative impacts commonly affect the patients and caregivers of HNC patients, manifesting as anxiety and depression [11].

The primary objective of this study is to perform a scoping review to chart the current literature on care challenges for HNC patients and caregivers in the United States, with a specific emphasis on caregiver burden, financial challenges, and nutritional toxicity experienced by patients. The secondary goal is to characterize the present status of qualitative literature in this domain and identify the researchers involved and the methodologies employed. To our knowledge, this scoping review represents the first attempt to contribute to this understanding by furnishing a comprehensive overview of the existing literature on the care challenges confronted by HNC patients and their caregivers in the United States.

## Review

Methods

A scoping review was conducted following Arksey and O'Malley’s methodology [12]. A scoping review methodology was selected to explore the body of literature on the challenges faced by patients with HNC and their caregivers. This approach allows us to comprehensively assess the volume of published studies, providing a clear overview. Our aim is to analyze the existing literature by focusing on three key aspects. First, we seek to identify prevalent themes that emerge across studies. Second, we examine the various methods used to deliver these programs. Finally, we explore the sociodemographic characteristics of the participants to gain a deeper understanding of the populations these programs serve.

Information sources and search strategy

A comprehensive search of several databases from each database's inception to October 2023 was conducted in compliance with the Preferred Reporting Items for Systematic Reviews and Meta-Analyses Extension for Scoping Reviews (PRISMA-ScR) guidelines [13]. The databases included Ovid Medical Literature Analysis and Retrieval System Online (MEDLINE) and Ovid Excerpta Medica Database (EMBASE). The search strategy was designed and conducted by an experienced librarian with input from the study's principal investigator. Controlled vocabulary supplemented with keywords was searched for head and neck neoplasms, financial stress, food insecurity, cost of illness, and caregiver burden in adult patients. The actual strategy listing all search terms used and how they are combined is available in Table 1.

**Table 1:** Database search strategy used for scoping review

Search Terms [PubMed]
(("Head and Neck Neoplasms"[Mesh] OR "Squamous Cell Carcinoma of Head and Neck"[Mesh]) OR ((head OR neck) AND (cancer OR neoplasm OR carcinoma OR tumor))) AND ("Financial Stress"[Mesh] OR "financial stress" OR "financial toxicity" OR "Caregiver Burden"[Mesh] OR "caregiver burden" OR "Housing Instability"[Mesh] OR "housing instability" OR "Food Insecurity"[Mesh] OR "food insecurity" OR "Cost of Illness"[Mesh]) Filters: English Sort by: Publication Date
Search Terms [Embase]
((('head and neck tumor'/exp OR 'head and neck neoplasms' OR 'squamous cell carcinoma of head and neck') OR ((cancer OR tumor OR neoplasm OR carcinoma) AND (head OR neck))) AND ('financial stress'/exp OR 'financial distress'/exp OR 'caregiver burden'/exp OR 'housing instability'/exp OR 'food insecurity'/exp OR 'cost of illness'/exp OR 'financial stress' OR 'financial toxicity' OR 'caregiver burden' OR 'housing instability' OR 'food insecurity')) AND [embase]/lim NOT ([Embase]/lim AND [Medline]/lim) AND [English]/lim

Selection criteria

**Figure 1:**
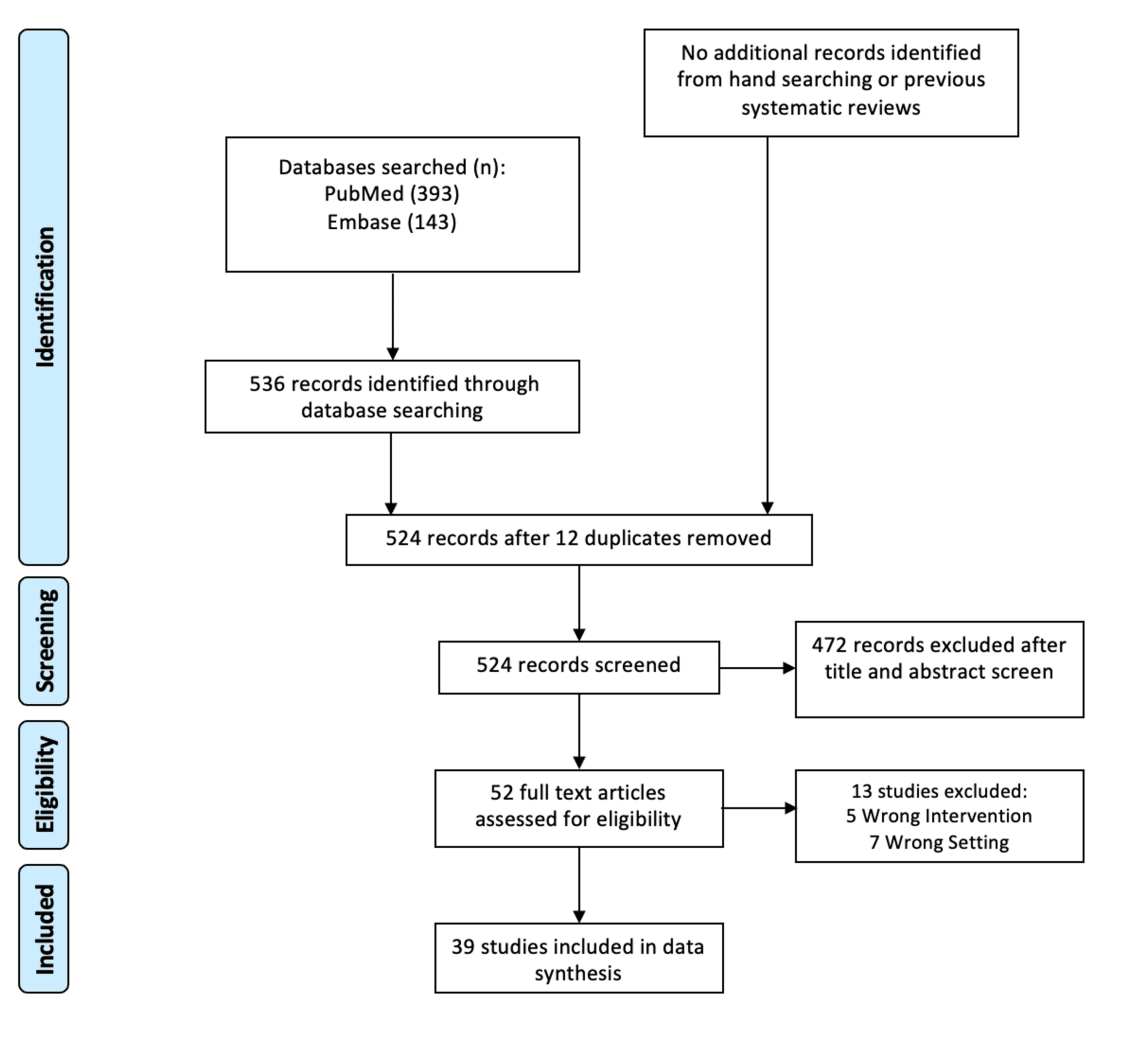
Flowchart of included studies

Studies meeting the following criteria were included in the analysis: (1) if the study's objective was to assess the impact of HNC on care provision for patients aged over 18 by either the patients themselves or the caregivers or if it provided a description of these needs; (2) if the publication was a full-text original article; (3) if the article was published in English; (4) if the article was published in a peer-reviewed journal; (5) if the study was conducted in the United States; and (6) if the full text was accessible. These criteria were applied to the initial 822 results, resulting in 280 eligible articles after title and abstract review by two independent reviewers (S.F. and D.K.) using Covidence (www.covidence.com). Following a subsequent full-text screening, 40 articles that met the inclusion criteria were included in the review. Figure 1 shows the PRISMA-ScR flowchart of the study describing the selection procedure.

Data extraction

The study involved the extraction of data from included articles, focusing on study design, respondent characteristics, outcome measures, and findings related to the influence of HNC on the capacity of patients and caregivers for self-care or caregiving. The identified data were organized into distinct domains, including caregiver burden, financial toxicity, and food insecurity. Consensual agreement on the distribution of collected data was achieved through collaborative efforts between two researchers (S.F. and A.S.).

Results

The initial literature search of the electronic databases yielded 822 studies. After removing duplicates, the articles were screened for inclusion and exclusion criteria, and 53 studies were retained for full-text review. Of the 40 studies, 23 were descriptive surveys, 8 were retrospective chart reviews, 6 were cross-sectional studies, and 4 were prospective cohort studies. All the included studies were single institutional studies.

Caregiver burden

Six studies investigated the impact of caregiving for individuals with HNC [14-19]. These studies collectively defined caregiver burden as a complex concept involving physical, psychological, emotional, social, and financial stressors faced by caregivers throughout the continuum of care, from acute treatment to survivorship. In a longitudinal prospective cohort study by Kudrick et al. involving 74 participants, the Caregiver Reaction Assessment (CRA) was utilized to measure caregiver burden [14]. Their findings indicated an association between patient employment status and caregiver health problems, with a notable increase in reported health problems among caregivers of nonworking patients by six months into treatment. Stavas et al. conducted a cross-sectional study and introduced the HNC Caregiving Task Inventory (CTI) to characterize various caregiving aspects, revealing a high overall burden despite low average difficulty in specific caregiving domains [17]. They identified medical care, symptom management, and psychosocial tasks as particularly challenging or discomforting for caregivers.

Maguire et al. also performed a cross-sectional study focused on the level of social support caregivers receive and their level of loneliness [16]. Caregivers provided sociodemographic information, impact of caring on their time and finances, as well as their level of social support (Oslo Support Scale), loneliness (3-point loneliness scale), and completed the Worry of Cancer Scale to measure fear of cancer. They found that caregivers who reported more loneliness, spent more time caring, and had greater financial stress from caring had higher scores on Worry of Cancer (Fear of Recurrence [FOR]). Female caregivers, those caring for younger survivors, and those with survivors who had undergone less extensive forms of surgery also reported higher FOR.

Nightingale et al. and Ramasamy et al. conducted interview-based surveys involving a total of 20 participants, utilizing the Burden Interview (BI) or Zarit Burden Interview (ZBI) to assess caregiver burden, alongside the Caregiver Quality of Life Index-Cancer (CQOLC) [15,18]. Nightingale et al.'s findings revealed an increasing trajectory of caregiver burden throughout HNC treatment phases, notably rising from treatment initiation to one month post-treatment, with correlations indicating a parallel decline in caregiver quality of life as patient well-being decreased [18]. Ramasamy et al. noted a proactive adoption of coping strategies by caregivers, where cultural beliefs and family played pivotal roles in managing and mitigating stress and burden on them [15]. Blood et al.'s assessment of 75 caregivers highlighted that male caregivers reported lower levels of strain and burden compared to their female counterparts, irrespective of other concurrent stressors [19]. Collectively, these studies underscore the intricate and evolving nature of caregiver burden in the context of HNC care, emphasizing the imperative for targeted support and interventions to address the challenges faced by caregivers.

Nutritional insecurity

Two studies focused on nutritional care for HNC patients, both underscoring the significant financial burden associated with HNC and its correlation with distinctive nutritional challenges these patients face [20,21] Given the potential for reduced food intake and malnutrition due to physical impediments from tumors, as well as issues such as odynophagia, dysphagia, toxicity from systemic treatments, metabolic dysfunction, and cachexia, HNC patients are evidently at substantial risk of food insecurity. Moreover, the specialized dietary needs inherent to HNC further compound the difficulty in obtaining sufficient nutrition, particularly considering the known financial strain associated with managing HNC, which could exacerbate challenges in accessing adequate dietary resources [20,21].

Berger et al.'s review of the National Health Interview Series (NHIS) from 2014 to 2018, involving 99,000 primary head and neck cancer (pHNC) patients, revealed a significant food insecurity rate of 17.7% within the HNC study population [20]. This proportion was notably higher (P = 0.026) than a previous national study on all cancer survivors from 2011 to 2014. Unlike findings in other cancer types, the study found no associations between gender, race, and Hispanic ethnicity and an increased likelihood of food insecurity in pHNC patients. The results underscore the distinct challenges faced by pHNC patients in terms of food security and advocate for routine screening as a crucial component of care to address these challenges and enhance nutrition, quality of life, and survivorship outcomes for pHNC patients. Alberda et al. conducted a descriptive qualitative study, focusing on nutritional insecurity in 10 HNC patients [21]. Through interviews, the study revealed the significance of addressing individual patient needs and tailoring recovery strategies, emphasizing detailed and innovative nutrition support featuring healthy and familiar foods. The study also highlighted the importance of considering patients' comorbidities and dietary restrictions. These findings underscore the necessity for patient-focused, evidence-based guidelines as a primary facilitator of nutrition care to ensure that all patients benefit from personalized nutritional support.

Financial toxicity

A majority of the studies included in this review focused on the financial burden placed on HNC patients, the factors that worsened financial status, and the feasibility of screening for financial toxicity. The most prominent factors that worsened financial toxicity included female sex [20-25], young age [23-26], having Medicaid [26], prior financial insecurity [22,25,27,28], cultural acclimation [23], advanced cancer stage [29,30], metastases [29], and unemployment [10,29].

Several studies aimed to characterize the relationship between financial toxicity and psychological burden or health-related quality of life [31-35]. Mongelli et al. found that patients with higher financial toxicity often reported increased psychological burden along with higher rates of anxiety and depression [36]. Three studies found that factors contributing to increased psychological burden included higher out-of-pocket costs, younger age, lack of insurance, and decreased wealth [27,32,36]. Broekhuis et al. reported that patients with protective financial factors such as a full-time job, private insurance, or an income above $100,000 still experienced increased psychological burden due to financial toxicity [34]. Apart from psychological burden, two studies characterized the effects of financial toxicity on overall health-related quality of life [35,37]. These studies found significantly decreased health-related quality of life in patients who experienced higher financial toxicity. Overall, these studies highlighted an association between higher financial toxicity and worse mental health outcomes.

Bensimon et al. and Massa et al. found that unemployment among HNC patients was a major contributor to financial burden, especially for caregivers [22,38]. This ultimately requires caregivers to choose between work and fully supporting HNC patients, further worsening HNC-associated financial toxicity [10]. The negative outcomes of financial toxicity include emotional stress regarding long-term financial status [25,31] inability to provide basic necessities for their families, and diminished personal savings [25,39].

Five studies found that a significant portion of HNC patients and caregivers experienced financial toxicity prior to chemotherapy and radiation therapy [22,25,27-29]. While most patients did not report a significant increase in financial burden during treatment, a quarter of them associated their increased financial toxicity with hospitalization during treatment [28]. Patients who reported greater financial toxicity after treatment also reported decreased satisfaction with their treatment and more missed treatment sessions [29] Jiang et al. found that worsening financial toxicity was a predictor for noncompliance to radiotherapy treatment [28]. Another study found that financial toxicity impaired patients’ ability to access and adhere to treatment regimes, unless to the detriment of their essential needs [10]. Jurica et al. reported that failure to adhere to medical treatments further worsened financial burdens on HNC patients [39] However, Lam et al. reported that HNC patients with greater financial toxicity were less likely to experience a delay in treatment [26]. One study stated that 69% of HNC patients relied on at least one cost-coping strategy to manage their healthcare-associated financial stress [36]. In general, these studies highlight the immense negative impacts of financial toxicity on the treatment and survival of HNC patients, especially those in lower economic populations.

Nguyen et al. found that respondents reported uncertainty about their financial future and worry about the amount of contribution they could make to patient care [31]. Day et al. recorded a large portion of HNC patients who felt that financing counseling would decrease their chances of experiencing financial burdens [40]. Farrugia et al. reported that financial counseling was found to be associated with decreased financial difficulty [41]. Patel et al. and Karukonda et al. reported a correlation between healthcare-associated financial toxicity and a lack of insurance knowledge, surveying patients using the Health Insurance Literacy (HIL) scale to measure their understanding of insurance plans and the services they covered [25,42]. Both studies found that patients were unaware of the financial costs of HNC prior to treatment, had limited understanding of insurance policies, and stated that educational materials on HNC costs were helpful long term. Some of these materials included information on the types of services included in radiation therapy, how to select an insurance plan that suited their situation, and how to best use their insurance plan [26,42].

Additionally, Fischer et al. and Barrows et al. found that provider education could significantly improve the ability for healthcare providers to assist their patients in mitigating financial toxicity [30,43]. In one study, financial counseling was found to be associated with lower financial toxicity [44]. Two studies reported attenuated financial burden when patients were provided with appropriate financial counseling and support resources. Five studies emphasized the need and benefit of early systematic screening of HNC patients and caregivers for financial toxicity risks [45]. Early intervention may play a crucial role in the patient education, support, treatment, and survival, allaying financial toxicity and improving the quality of life of HNC patients and their caregivers.

Discussion

HNC presents a significant health threat in the United States, requiring a complex management approach that involves multidisciplinary interventions such as oncological and surgical procedures, drug management, and psychological support [46,47]. For patients, the physical and financial impact of HNC, coupled with barriers to comprehensive care, can significantly affect their quality of life and self-esteem [35,37]. Caregivers often bear the weight of caregiving responsibilities, and a lack of sufficient support can contribute to diminished psychological well-being, leaving them with a sense of isolation [14-19]. The findings of this scoping review highlight the significant challenges faced by HNC patients and their caregivers.

The challenges faced by caregivers, as shown in this review, are multifaceted and require a broad approach when combating them [48]. Though there are interventions actively being made to improve the quality of life of HNC patients, the issues of caregivers tend to take a back seat during discussions of patient care. Caregivers experience many hardships that greatly impact their lives such that they could be considered a silent patient [14,15,18]. Though their burdens are secondary issues caused by HNC, their worries are equally important. Findings in the studies included in this scoping review highlighted concerns about the mental health of caregivers, noting a decline in their well-being as the patient's condition worsened [15,17,18]. On the other hand, an increase in age was found to be inversely related to caregiver burden, indicating that older caregivers tended to feel less burden compared to their younger counterparts [14]. We hypothesize that this might be attributed to the older caregivers having achieved greater financial and family stability, along with being better equipped with coping mechanisms compared to their younger counterparts.

As studies have shown, a significant portion of caregivers felt a lack of support and educational guidance regarding financial dealings [14,17,39]. Research on caregiver burden would benefit from centering on community resources, counseling, and educational programs [17]. Additionally, caregivers lack the support needed outside of the hospital to maintain their household. Many caregivers are women who unexpectedly become the primary earner of their household. This only adds to the burden on their shoulders as they attempt to balance patient care alongside their own lives [17]. The responsibilities assigned to these caregivers often exceed the capacity of a single individual, potentially leading to a long-term negative impact on patient care, as patients may lose their primary support. This challenge is further exacerbated by cultural and socioeconomic factors. Therefore, when exploring potential solutions, it is crucial to consider the cumulative effects of these issues. One limitation of studies focusing on caregiver burden was that many of them concentrated on the short-term impacts of HNC, neglecting consideration of long-term effects such as emotional burdens, diminished financial status, and an overall decrease in quality of life.

Studies included in this review that focused on nutritional care emphasized the heightened risk of food insecurity among HNC patients [20,21]. Factors contributing to this risk include reduced food intake, malnutrition, physical impediments from tumors, odynophagia, dysphagia, toxicity from systemic treatments, metabolic dysfunction, and cachexia [7,18]. Berger et al.'s review of the National Health Interview Series (NHIS) identified a significant food insecurity rate of 17.7% among pHNC patients, higher than that reported in a previous national study on all cancer survivors [20]. The results emphasize the distinct challenges faced by pHNC patients in terms of food security, with no observed associations between gender, race, and Hispanic ethnicity. Alberda et al.'s qualitative study focused on nutritional insecurity in HNC patients, stressing the importance of addressing individual patient needs, tailoring recovery strategies, and providing personalized nutrition support based on patient comorbidities and dietary restrictions [21]. Overall, the findings underscore the necessity for routine screening and patient-focused, evidence-based guidelines to enhance nutrition, quality of life, and survivorship outcomes for HNC patients.

Along with increased stress on caregivers and nutritional instability, head and neck cancer impose a significant financial burden on many patients. As mentioned in this scoping review, characteristics such as younger age of the patient, lower income, unmarried status, and unemployment were all associated with greater risk for financial toxicity [25,28,29,41,49]. Additionally, some patients reported high financial burden due to loss of earnings after diagnosis. Even patients with protective factors against financial toxicity experienced increase financial burden [34]. This financial toxicity was often accompanied by increased strain on patients’ and caregivers’ mental health and reported quality of life [37,49].

These results highlight a relationship between increased financial toxicity and deteriorating mental health/quality of life of both patients and their caregivers. Our scoping review highlights that a substantial number of HNC patients and caregivers encountered financial toxicity before initiating chemotherapy and radiation therapy [22,25,27-29]. Patients who reported higher financial toxicity post-treatment also indicated reduced satisfaction with their treatment and a higher incidence of missed treatment sessions, serving as a predictor for noncompliance with radiotherapy treatment [29,28].

Multiple studies explored factors that could mitigate the negative effects of financial toxicity and reported that educational material concerning costs associated with treatment would be helpful in equipping patients and their families to better face financial challenges [39,41,44]. In addition to educating patients, educating healthcare providers about financial toxicity and resource limitations could help reduce the financial burden on patients and their families. [43]. Collectively, these studies point to the importance of education on financial toxicity for both patients and their providers. Educating patients and providers on financial toxicity could help mitigate downstream effects of financial toxicity, such as lower quality of life and psychological strain. Interventions targeting education on financial toxicity warrant further exploration. Summary of findings from the included studies is presented in Table 2.

**Table 2:** Summary of findings from the included studies COST, COmprehensive Score for financial Toxicity; CRA, Caregiver Reaction Assessment; HNC, head and neck cancer; SSS, Social Support Survey

Author	Year	Study Design	Focus	Study Aim	Sample Size	Outcome Measures	Summary Findings
Mott et al. [9]	2022	Retrospective chart review	Financial toxicity	To characterize financial hardship in the psychological response and coping behaviors domains in patients with HNC and those with other cancer	n=357,052 (HNC), 21.4 million (other)	Financial hardship in the psychological response, coping behaviors domains	Patients with HNC reported greater levels of coping behaviors hardship than those with other types of cancer. Patients with HNC demonstrated similar psychological financial hardship as those with other kinds of cancer.
Jella et al. [10]	2021	Cross-sectional	Financial toxicity	To understand the economic burden imposed by HNC diagnoses essential to contextualizing decision-making for the patients	n=710	US National Health Interview Survey assessed patient-reported financial insecurity	Insurance and poverty status were significantly associated with difficulty paying medical bills. HNC patients may experience financial burden due to both out-of-pocket health costs and other non-health related costs.
Kudrick et al. [14]	2023	Prospective cohort study	Caregiver	To evaluate the prevalence of and identify risk factors for caregiver burden in HNC survivorship	n=96	19-item SSS, CRA, and 3-item Loneliness Scale. Patient health-related quality of life was assessed using the University of Washington Quality of Life scale	Caregivers of nonworking patients reported higher CRA scores, indicating disarrayed schedules, financial toxicity, shortage of family support, and health issues. Female caregivers had significantly worsening scores on SSS. The proportion of lonely caregivers increased over the duration of treatment. Caregiver characteristics: 96% White, 50% female, 84% married or living with partner, 53% had been employed for 18 years or more.
Ramasamy et al. [15]	2021	Survey studies	Caregiver	To explore the psychosocial issues faced by the primary caregivers of advanced HNC patients with the primary objective to understand their experiences within social context	n=15	Zarit Burden Interview Schedule, Caregiver Quality of Life index-cancer	Most caregivers reported mild-to-moderate burden associated with caring for the patients. Most caregivers scored low on quality of life when assessed. Caregiver characteristics: 27% male, 74% female ,74% spouses, mean age 46.1.
Maguire et al. [16]	2017	Survey studies	Caregiver	To establish the role of care-related stressors as distinct from survivor characteristics in predicting fear of recurrence in HNC caregivers	n=180	Functional Assessment of Cancer Therapy Questionnaire to assess health and quality of life, Oslo Support Scale to assess social support, 3-point Loneliness Scale to assess loneliness, Worry of Cancer Scale to measure fear of recurrence	Caregivers reporting higher levels of loneliness, more time caring, and greater financial stress from caring had higher fear of recurrence. Caregivers whose survivors underwent less extensive treatment, those who were female, or those caring for younger patients reported high fear of recurrence. Caregiver characteristics: 24% male, 76% female, 73.4% spouses, mean age 57.3.
Nightingale et al. [18]	2014	Prospective cohort study	Caregiver	To explore the psychosocial functioning of 10 HNC patient-caregiver dyads over the radiation/chemoradiation treatment period	n=10		Quality of life decreased for many caregivers during the middle treatment. Caregiver burden increased over the course of treatment. Patient-caregiver dyads expressed interdependence in quality of life.
Blood et al. [19]	1994	Survey studies	Caregiver	To assess caregiver strain and burden	n=75		Caregiver strain and burden was inversely proportional to time since diagnosis. Male caregivers reported less burden than their female counterparts. Participants reported strain and burden independent of other stressors in their lives. Caregiver characteristics: spouses of individuals with laryngectomies.
Berger et al. [20]	2021	Survey studies	Nutrition	To determine the food security status of patients with a history of HNC and compare to other cancer types	n=199,000	International Health Interview Series to assess food security status	Food insecurity was significantly higher in pharynx/throat HNC than those with thyroid cancer. Food security in HNC patients showed no significant difference when categorized into gender, race, or ethnicity. Black patients were more likely to have food insecurity with colon or thyroid cancer. Hispanic patients were more likely to have food insecurity with thyroid cancer.
Alberda et al. [21]	2017	Survey studies	Nutrition	To understand the patients’ experiences with nutritional care in the context of their treatment and recovery	n=20	Semi-structured interview guide	Lack of coordination throughout care and conflicting messages from healthcare providers led to uncertainty, confusion, and isolation in HNC patients. Both HNC and esophageal cancer patients exhibited a need for patient-focused nutritional care with informal/formal support.
Ku et al. [23]	2023	Survey studies	Financial toxicity	To assess patient-reported financial toxicity prior to and after completion of radiation therapy, and uncover any interactions with socioeconomic factors, quality of life,	n=80	FACIT-COST and FACIT-TS-G surveys	Higher financial toxicity was associated with decreased age, thyroid primary disease, metastatic disease, Medicaid, Hispanic ethnicity, unemployment, and G-tube placement. Lower financial toxicity was associated with cutaneous primary disease and ability to speak English. Patients with worse financial toxicity reported lower satisfaction with care after radiation therapy.
Diao et al. [24]	2022	Survey studies	Financial toxicity	To describe the nature, extent, and predictors of financial toxicity in patients with oropharynx cancer after radiation therapy or surgery	n=400	MD Anderson Symptom Inventory Head and Neck, Neck Dissection Impairment Index, Effectiveness of Auditory Rehabilitation Scale, 8-question Financial Toxicity Instrument	Patients who had surgery without post-operative radiation had less short-term financial toxicity. Younger age, primary radiation therapy, female sex, Black non-Hispanic race, unmarried status, lower income, tonsil subsite, and worse scores on MDASI-HN, NDII, and EAR were associated with worse long-term financial toxicity. Overall, patients reported high levels of long- and short-term financial toxicity
Patel et al. [25]	2022	Survey studies	Financial toxicity	To assess health insurance literacy in patients with HNC	n=42	Health Insurance Literacy Measure Scale	A significant portion of patients reported limited understanding in their ability to choose, compare, understand, or use their health insurance.
Jiang et al. [29]	2020	Survey studies	Financial toxicity	To test the hypothesis that patients who experience more financial toxicity are more likely to miss radiation therapy appointments	n=164	EORTC QLQ C-30 assessed financial toxicity, noncompliance was measured by missing one or more appointments	Financial toxicity was significantly higher at the end of treatment than the beginning for patients that reported financial toxicity. Having Medicaid insurance and worsening financial toxicity were associated with treatment noncompliance.
Fischer et al. [30]	2020	Survey studies	Financial toxicity	To measure the success of a one-day curriculum aimed at educating fellow physicians on resources to combat financial toxicity for their patients	n=19	Cost-health Literacy Survey to evaluate impact of the one-day curriculum	After the one-day curriculum, significantly more fellow physicians agreed/strongly agreed that they could help patients experiencing financial toxicity. Provider education on financial resources for patients could help limit financial toxicity.
Nguyen et al. [31]	2023	Cross-sectional	Financial toxicity	To assess the feasibility of financial toxicity screening of HNC patients and their caregivers and to describe financial toxicity levels of HNC patients and their caregivers	n=36	Survey, COST scores measured financial toxicity	Members of the caregiver and patient groups both reported high financial toxicity. Caregivers reported high levels of concern about their financial health and future contributions to patient care.
Chen et al. [33]	2020	Survey studies	Financial toxicity	To determine the prevalence of financial hardship in Hispanic women with thyroid cancer	n=273	Short Acculturation Scale for Hispanics, Study-Specific Survey that assessed financial status, insurance status, and material measures of financial hardship	Low-acculturated women experienced high financial burden across all age groups. Financial hardship was decreased and decreased with age for high-acculturated women.
Broekhuis et al. [34]	2021	Survey studies	Financial toxicity	To characterize financial burden and decreased quality of life in patients with thyroid cancer over a 28-year period	n=147	Phone survey assessing financial burden and its related psychological hardship	Patients who reported financial burden were significantly impacted more by psychological financial hardship Financial burdens affect patients with and without protective financial factors
Kayser et al. [35]	2021	Survey studies	Financial toxicity	To analyze the lived experience of cancer patients’ financial hardship from diagnosis to post-treatment	n=26	Patient interviews assessed the experience of cancer patients’ financial hardship	Cancer survivors do not experience financial toxicity as an isolated process. Financial toxicity can vary based on age and life transitions.
Mongelli et al. [36]	2020	Survey studies	Financial toxicity	To investigate whether financial distress would be common in thyroid cancer survivors and would be associated with poor health-related quality of life	n=1,743	Financial Distress Questionnaire, Patient-reported Outcomes Measurement Information System (29-item)	A large proportion of thyroid cancer survivors reported financial difficulties. Financial difficulties were associated with worse anxiety and depression. Financial difficulties were associated with worse health-related quality of life in five patient-reported outcomes.
De Souza et al. [37]	2017	Survey studies	Financial toxicity	To prospectively estimate patient-centered financial stress and its relationship with healthcare utilization in patients with HNC	n=73	Study-specific survey collected demographics of the patients, healthcare utilization was measured by hospital admissions and outpatient appointments, logistic regression models identified factors associated with using coping strategies	A majority of patients relied on at least one cost-coping strategy. Decreased wealth and higher out-of-pocket costs were associated with using cost-coping strategies. Perceived social isolation was associated with non-adherence to medicine, missing appointments, and more cost-coping strategies.
Massa et al. [38]	2022	Retrospective chart review	Financial toxicity	To describe the total and out-of-pocket costs associated with HNC survivorship and the risk for financial toxicity among this population	n=19,098	Difference between a patients’ mean monthly survivorship costs, mean monthly baseline costs	Female sex, multimodal therapy, and hypopharyngeal tumors were associated with increased total and out-of-pocket costs. Multimodal therapy was associated with higher costs compared to radiation or surgery alone.
Jurica et al. [39]	2021	Prospective cohort study	Financial toxicity	To quantify the financial toxicity in head and neck squamous cell carcinoma patients that experienced treatment delays	n=438	Financial toxicity was calculated using hazard ratios, pembrolizumab cost, and dosing data	Failure to meet established treatment guidelines leads to significant institutional financial toxicity
Day et al. [40]	2020	Survey studies	Financial toxicity	To seek the perspectives of HNC patients who underwent surgery on cost of care conversations	n=19	COST-FACIT assessed financial toxicity, 32-question survey assessed experiences with cost of care conversations	All patients whether insured or uninsured preferred discussing costs before treatment. Most patients preferred to speak to an insurance agent or financial advisor rather than their surgeon about the costs of care. Medicaid/Uninsured patients experienced the most financial toxicity.
Farrugia et al. [41]	2021	Retrospective chart review	Financial toxicity	To investigate the financial impact of financial counseling on financial toxicity in patients with HNC	n=387	EORTC QLQ C-30 to detail financial difficulty	No significant difference was found for financial toxicity between patient preoperative score to postoperative scores. Financial counseling was found to be associated with decreased financial difficulty.
Karukonda et al. [42]	2021	Survey studies	Financial toxicity	To assess financial toxicity, patient-reported outcomes, and attitudes on the role of cost in treatment decisions	n=60	Study-Specific Surveys to collect demographics and cancer details, COST score to assess financial toxicity	A majority of patients felt that education about the costs associated with their care would be helpful. A large proportion of patients had a COST score indicating at least borderline financial toxicity.
Barrows et al. [43]	2020	Survey studies	Financial toxicity	To estimate the comparative prevalence of financial and psychological hardship among thyroid and non-thyroid cancer patients in the United States	NR	Cancer Self-Administered Questionnaire, Medical Expenditure Panel Survey	Thyroid cancer survivors reported more psychological financial burden, material financial hardship, and concurrent material and psychological hardship than non-thyroid cancer survivors. Younger age and lack of insurance were associated with psychological financial hardship.
Ma et al. [44]	2020	Retrospective chart review	Financial toxicity	To investigate the association between financial well-being and survival outcomes in patients with HNC	n=284	Self-reported financial difficulties	Patients with reported financial difficulties experienced worse overall and cancer-specific survival
Jacobson et al. [46]	2012	Retrospective chart review	Financial toxicity	To investigate the costs associated with oral cavity, oropharyngeal, and salivary gland cancer	n=6,812	Direct medical expenditures, indirect expenditures were measured by short-term disability days	Oral cavity, oropharyngeal, and salivary gland cancer patients with Medicare, Medicaid, or commercial insurance had higher healthcare costs. Patients receiving surgery, radiation, and chemotherapy had the highest costs of care.
Baddour et al. [49]	2021	Cross-sectional	Financial toxicity	To document objective financial metrics and their impact on subjective financial toxicity in HNC survivors	n=71	COST score and Financial Distress Questionnaire assessed patient-reported financial toxicity	Worse COST scores and financial distress were associated with lower earnings, loss of earnings after diagnoses, and greater annual out-of-pocket expenses
Genther et al. [50]	2012	Cross-sectional	Financial toxicity	To determine the association between safety-net hospital care and short-term outcomes after head and neck surgery	n=123,662	Associations between safety-net burden and short-term mortality, medical/surgical complications, length of hospitalization, costs	Safety-net hospitals can provide specialty care without increased complications or costs

Knowledge gaps

The literature underscores a noticeable void in published studies regarding nutritional deficiency, while the predominant focus of existing research centers on the financial challenges faced by patients and their families, coupled with the caregiver burden. Though they provide insightful views on the issues faced and the possible interventions, none provide an overall view incorporating the negative impacts that HNC places on nutrition, psychological health, and financial status. Some studies focus on the improvement of patient care, assuming that quicker resolutions will decrease the hardships faced by caregivers. However, this approach fails to acknowledge that many of the burdens continue even after treatment, such as the financial and psychological toll of care for a sick loved one.

Strengths and limitations

All things considered, our review focuses on the interrelation between the various stressors HNC caregivers experience throughout treatment and patient care. It accounts for socioeconomics, screening availability, literacy, and how they can deter positive outcomes as a whole. Additionally, it analyzes how the stated subfactors tie into the psychological, nutritional, and financial burden of HNC patients and caregivers. However, a larger and more diverse population is needed for a more in-depth depiction of the common challenges faced by the HNC community.

The definition of financial toxicity was left to the interpretation of the reviewers. Thus, this review may have excluded healthcare-associated financial circumstances that constitute burden on HNC patients. The selection criteria limited the number of papers in this review. Only two peer-reviewed sources were analyzed for this review. Thus, papers relevant to this review may have been excluded due to selective sourcing. The papers in this study were required to be based in the United States and written in English, which limits the studies that are included in the paper.

## Conclusions

This scoping review reveals gaps in the literature regarding care challenges for HNC patients and caregivers. While financial challenges are well-documented, more research is needed on nutritional insecurities and caregiver burdens. Caregivers face significant stressors, emphasizing the need for targeted interventions and research addressing their specific needs.
