# Correction: Uncovering Care Challenges for Head and Neck Cancer Patients and Caregivers in the United States: A Scoping Review

**DOI:** 10.7759/cureus.c249

**Published:** 2025-08-15

**Authors:** Sharon Amole, Roger M Roe, Soroush Farsi, Dang-Khoa Nguyen, Emily Hallgren, Lauren Tong, Deanne King

**Affiliations:** 1 Department of Otolaryngology – Head and Neck Surgery, University of Arkansas for Medical Sciences, Little Rock, USA; 2 Department of Internal Medicine, University of Arkansas for Medical Sciences, Fayetteville, USA

Author Emily Hallgren’s affiliation was incorrectly listed as ‘Department of Otolaryngology – Head and Neck Surgery, University of Arkansas for Medical Sciences, Little Rock’.

This has been corrected to ‘Department of Internal Medicine, University of Arkansas for Medical Sciences, Fayetteville’.

